# Assessing the Efficiency of Bin-Centered Solid Waste Segregation System in Ashanti Region, Ghana

**DOI:** 10.1155/tswj/6169623

**Published:** 2025-06-27

**Authors:** Lyndon N. A. Sackey, David Nii Ayi Anum, Ekua Yekowah Ampiah, Charlotte Adwoa Marfo, Kodwo Amos, Nana Ama Antwiwaa Ahorlley, Lawrencia S. Y. Agyemang, Hamlyn Samuel Tetteh Addy

**Affiliations:** Department of Environmental Science, Faculty of Biosciences, College of Science, Kwame Nkrumah University of Science And Technology, Kumasi, Ghana

**Keywords:** Ghana, organic waste, plastic waste, segregation, waste management

## Abstract

Kwame Nkrumah University of Science and Technology (KNUST) established a waste segregation system that provided plastic waste to feed a plastic recycling facility to improve solid waste management in 2017. However, since the establishment of the segregation system, there has not been any assessment to ascertain its efficiency. Hence, this research seeks to assess the efficiency of KNUST's waste segregation system. The study utilized a quantitative research approach method to assess the efficiency of the segregation system. A total of 500 randomly selected participants, including students, lecturers, administrators, cleaners, and other staff, participated in the survey. Also, to ascertain the efficiency of the segregation system, through stratified random sampling, 67 bins were selected for the segregation of plastic waste. The results indicated poor attitudes towards segregation, low adherence, and ineffectiveness. Analysis of the waste components generated on campus revealed that 64.7% of the participants generated paper as part of the total waste produced, a substantial 83.5% generated plastics, 82.5% generated food waste, and 28.2% generated tins/cans. A corresponding 9.4%, 11.24%, and 4.4% generated fiber bags, glass, and other waste types, respectively. The plastic waste stream consists of polyethylene terephthalate (PET), high-density polyethylene (HDPE), low-density polyethylene (LDPE), and polystyrene (PS). Of the participants, 43.9% knew about waste segregation on campus, and 75.5% had seen the bins. Of the participants, 56.5% knew what the color codes of the bins meant. Barriers to effective segregation included low publicity, insufficient bins, and low monitoring. Management should intensify publicity, introduce more bins, and diversify the system.

**Summary**

• The plastic waste stream contained polyethylene terephthalate (PET), high-density polyethylene (HDPE), low-density polyethylene (LDPE), and polystyrene (PS).
• Poor attitude toward the waste segregation system on campus.
• PET and LDPE were the highest plastic waste.
• Less than half of the respondents had an idea about the segregation system.

## 1. Introduction

Waste refers to products or substances no longer suitable for their intended use, often used as food or reactants in natural ecosystems. Human waste materials are highly resilient and take a long time to disintegrate [1]. Waste can be any valuable resource, like time, energy, or materials, that has been lost or diminished in value [2]. International agencies and bodies have different opinions on waste, with Article 5 of the Basel Convention defining it as substances or objects disposed of or intended to be disposed of by national law, and the Organization for Economic Cooperation and Development (OECD) defining it as substances or objects, except for radioactive materials, requiring disposal or recovery by national law [3].

Sustainable solid waste management is crucial for both developed and developing nations, as it involves efficiently managing large volumes of waste to preserve the environment and ensure human survival. While the biodegradability of organic municipal solid waste (MSW) may not be a major issue, nonbiodegradable plastic waste poses a threat to the environment, as it can linger for 20 to 1000 years [4]. So when plastic waste is segregated, it can help in recycling it in a better way and protect the environment and human health [5].

Waste management involves collecting, transporting, processing, recycling, disposing, and monitoring waste materials to minimize their impact on health and the environment [6]. In Ghana, about 12,710 tons of solid waste are produced daily, with only 10% being collected and disposed of at appropriate sites [7]. The main challenge in Ghana's solid waste management is the labor-intensive and ineffective collection and disposal of waste, which accounts for 33% of global solid waste production. Urban waste management in Ghana is a significant challenge due to the daily volume of waste and the lack of efficient strategies [8, 9]. City authorities spend around 6.7 million cedis (3.4 million US dollars) annually on waste collection and transportation and pay garbage contractors to maintain landfills [10, 11]. This indiscriminate waste disposal leads to poor sanitation, resulting in an estimated 290 million dollars spent on waste management annually, which represents 1.6% of the nation's GDP. Landfills are the primary method used in the Kumasi metropolis to manage solid waste, but their capacity is depleting quickly due to the lack of waste segregation [11].

Waste segregation is a crucial step in waste management, enabling optimal reuse, recycling, and recovery (3Rs) [12]. It involves sorting and separating waste types for easier recycling and disposal. However, it has received little attention in developing countries due to a lack of awareness, poor legal frameworks, economic motivation, and low planning priorities [12, 13]. In Ghana, proper solid waste management has become a critical environmental issue. According to Armijo De Vega et al. [14], waste reuse, recycling, and recovery (3Rs) can reduce waste disposal by up to 65% if well-planned and managed. Waste should be segregated based on its type, treatment, and disposal methods. Organizing waste helps identify recyclable products, minimize waste generation, and set aside reusable goods. Proper waste segregation reduces landfill waste, lowers environmental and human health costs, and is essential for public health. In Ghana, efforts to remediate solid waste management have become a critical environmental issue for its Metropolitan, Municipal, and District Assemblies [15]. Al-Khatib et al. [16] reported that the plastic fraction (10.1%) is mainly composed of LDPE films (39.8%), PET bottles (21.9%), HDPE rigid (19.0%), and polypropylene (PP) rigid (11.5%), all of which are technologically recyclable. Saadeh et al. [17] indicated that 80% of the companies supported the social strategies that implement community awareness programs. Almost 92% of the companies agreed with legal strategies that enforce legislation to support PW recovery and recycling practices. Adefris et al. [18] highlight the need to focus on awareness-raising efforts among the general public in order to improve the knowledge, attitudes, and behaviors of individual households and residents toward solid waste segregation practices. Dharia et al. [5] reported that the proposed source segregation bin results in high recyclability of household waste, and use of it has no environmental impact caused by waste. Fadhullah et al.'s [19] study found that the waste segregation practice among respondents can be considered low, where the number of respondents who segregate their waste was equivalent to those who did not, which implies there is room for improvement.

Kwame Nkrumah University of Science and Technology (KNUST) has implemented a waste segregation initiative by introducing 332 color-coded bins at strategic locations across campus. The bins are labeled yellow for plastic waste and green or black for other waste. The bins are strategically placed throughout the university campus. The KNUST recycling plant is fed by the plastic waste gathered in the yellow bins. The facility is an integrated plastic recycling plant and handles HDPE, PP, and LDPE. The recycling machine can crush and pelletize plastics. It has a 2-ton capacity for the pelletizer and 5-ton capacity for the crusher per day.

Waste segregation is a crucial method for dividing waste into different compositions and helps to easily add value to solid waste [7]. The segregation system helps in understanding the waste stream and assists in value addition. The KNUST campus implemented a solid waste segregation system to supply the campus plastic recycling plant with plastic waste. However, the efficiency of the segregation has not been assessed since its implementation. Hence, this research seeks to assess the efficiency of KNUST's waste segregation system. To address this, this research is being conducted to investigate residents' attitudes toward waste segregation and collect data on its effectiveness and understanding. Addressing these issues was essential for improving the waste segregation system on the KNUST campus and possibly across the nation.

The assessment will help stakeholders identify and address loopholes, providing a baseline for waste stream analysis and enabling projections for campus recycling sustainability. The data will also aid the government and policymakers in designing waste segregation systems for a larger population and the country. An effective system will help the country achieve Sustainable Development Goal 12 by reducing, reusing, and recycling waste.

## 2. Materials and Methods

### 2.1. Study Area

**Figure 1:**
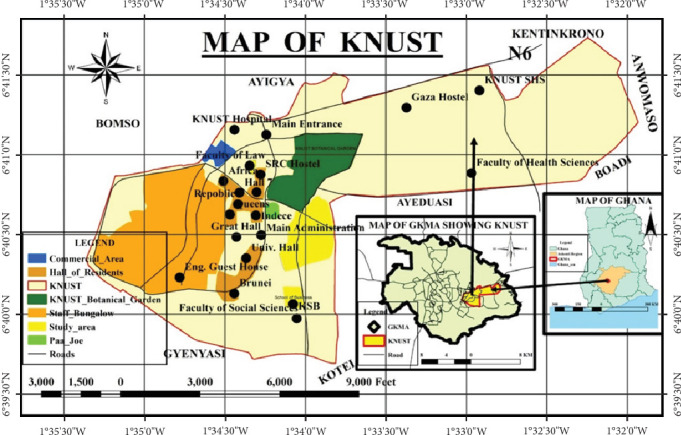
Map of study area.

KNUST is a public university in Ghana focusing on science and technology. The university covers a total land area of 2512.96 acres. The main campus, about 7 mi^2^ in area, is about 13 km east of Kumasi (Figure 1), the Ashanti regional capital. The campus is flanked by suburbs like Bomso, Ayigya, Kentinkrono, Ayeduase, and Kotei. The main campus consists of several standing facilities: halls of residence, hostels, an administration building, a college area, a commercial area, botanical gardens, sports complexes, markets, and staff bungalows.

### 2.2. Research Design

The study utilized a mixed approach to investigate the perceptions of university community residents about the segregation system on the KNUST campus. It collected qualitative data through interviews, observations, and analysis of primary and secondary sources. The waste disposed of in the bins was also characterized to ascertain the effectiveness of separation. This approach was chosen to provide a comprehensive understanding of the context and address challenges related to monitoring the segregation system. The mixed approach effectively addressed the challenges arising from the study, allowing the researcher to better understand the impact of bin-centered segregation on the KNUST campus.

### 2.3. Research Approach

The KNUST campus has implemented a bin-centered waste segregation system to separate waste from plastics for recycling. This study has multiple objectives: (1) using an exploratory approach: investigating the efficiency of this system and aims to improve its effectiveness. The exploratory approach aims to develop a better understanding and evaluation of the system, which has not undergone substantial assessment before this study. Also, (2) quantitative approach: This research aims to gather data on the types and quantities of plastic materials used on campus, using a quantitative approach to collect information for quantification and statistical treatment to support or refute “alternate knowledge claims” [20]. (3) Hypothesis testing: hypothesis testing of the knowledge claim (about color codes, adherence to segregation, and attitude towards segregation).

### 2.4. Sampling Technique

**Figure 2:**
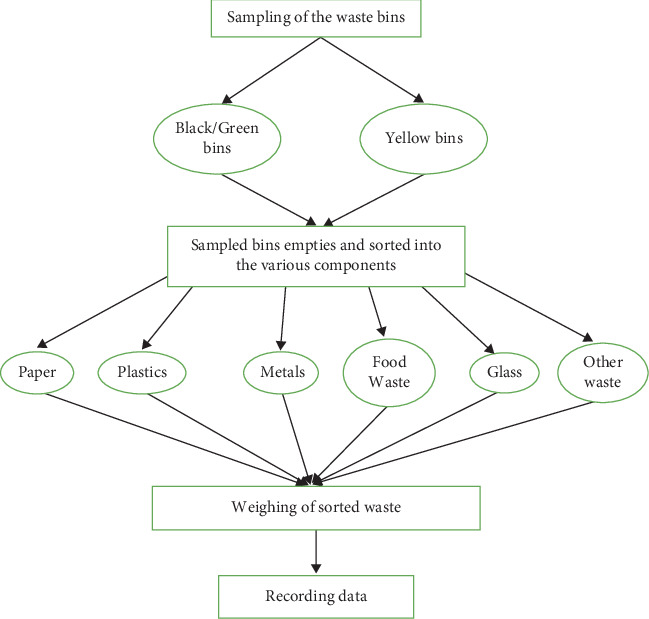
Flow chart of sampling procedure.

The study used probability sampling, specifically stratified random sampling, to categorize university community members based on their occupations (administrators, lecturers, workers, students, and cleaners). The research team was stationed at frequent campus routes, collecting samples from car routes, footpaths, motorcycles, and bicycle paths randomly from all the abovelisted university members. The selected sample plastic bins were emptied and sorted, with the weight of plastics measured against all other wastes in yellow-coded bins and poured back inside and vice versa (Figure 2).

In addition, the American Society for Testing and Materials (ASTM) method was used to sort MSW, a waste material that involves the collection, transport, processing, and disposal of waste materials for health, environmental, and aesthetic purposes. This method is crucial for local government authorities and industrial plants and laboratories. The test technique involves manually sorting MSW samples over a week to determine their composition. The steps include gathering a representative sample of unprocessed garbage, sorting the waste into parts, data reduction, and reporting the results. These waste management standards are essential for local government authorities and industrial plants and laboratories.

### 2.5. Sources of Data

The study utilized primary and secondary sources to collect data on waste segregation on the KNUST campus, utilizing questionnaires and color-coded bins for participant input and real-time insights for accurate and timely information. The study utilized secondary data from existing literature, internet, books, and peer-reviewed journals to understand waste segregation, providing a foundation for the entire research, which served as a stepping stone in the process.

### 2.6. Target Population and Sample Size

The study targeted all KNUST campus residents, with a student population of over 80,000 as of 2022, according to the acting university relations officer, Mr. James Oberko. A total of 500 randomly selected participants, including students, lecturers, administrators, cleaners, and other staff, participated in the questionnaires using Slovin's formula. But here, looking at the nature of the population, 80% of the estimated sample size, 100, was added to the 400 calculated through Slovin's formula.

Furthermore, the study was conducted on 33 randomly selected yellow-coded plastic bins and a similar number of green/black-coded bins, recording the plastic weights and other waste fractions. The sample size of the waste bins used for the research was derived from 20% of the total number of bins on campus. The exercise was conducted twice a week for 3 weeks, ensuring accurate measurements of waste fractions. Also, the research focused on waste bins in the University's main campus, hall of residence, commercial area, and bungalows. Out of 332 bins, 66 waste bins were sampled from lecturer's bungalows, commercial areas, colleges, and the great hall for data collection, as it provides a fair representation of the total bins on campus.

### 2.7. Data Collection Instrument

This study used field notebooks, hardcopy questionnaires, and Google Forms for data collection. Waste bins were segregated using a weighing scale (F1976), with yellow-coded and well-labeled waste poured onto a polythene spread, separated into plastic and other waste classes using hand gloves and nose masks. Green/black-coded and well-labeled waste was weighed separately. Plastic and other waste fractions were expressed as a percentage of their combined total to assess segregation efficiency on the KNUST campus.

The waste was sorted into various categories, such as PET, PVC, and PS, and weighed individually and the total weight was then taken using a scale. The weighing scale was calibrated before each new measurement to ensure data accuracy.

Gloves were used to protect hands from bin dirt, nose masks to shield noses from foul scents, and inhalation of obnoxious chemicals and gases.

The following equations were used to derive the waste percentages associated with plastic and all other waste fractions in the 2 bins shown in Equations (1), (2), (3), and (4).

(1) $$\begin{array}{c} \%\text{YB}=\frac{\text{WPYB}}{\text{TWYB}}, \end{array}$$

where YB is % plastics in the yellow-coded bin, WPYB is the weight of plastics in the yellow-coded bin, and TWYB is the total waste weight in the yellow-coded bin.

(2) $$\begin{array}{c} \%\text{OWYB}=\frac{\text{WOWYB}}{\text{TWYB}}, \end{array}$$

where OWYB is % other waste in the yellow-coded bin, WOWYB is the weight of all other wastes in the yellow-coded bin, and TWYB is the total waste weight in the yellow-coded bin.

(3) $$\begin{array}{c} \%\text{OWGBB}=\frac{\text{WOGBB}}{\text{TWGBB}}, \end{array}$$

where OWGBB is % of all other wastes in the green/black-coded bin, WOGBB is the weight of all other wastes in the green/black-coded bins, and TWGBB is the total waste weight in the green/black-coded bin.

(4) $$\begin{array}{c} \%\text{PGBB}=\frac{\text{WPGBB}}{\text{TWGBB}}, \end{array}$$

where PGBB is % of plastic in the green/black-coded bin, WPGBB is the weight of plastics in the green/black-coded bins, and TWGBB is the total waste weight in the green/black-coded bin.

The percentage compositions of individual categories of plastics were calculated according to Equation (5).

(5) $$\begin{array}{c} P_{i}=\frac{W_{i}\times 100}{W_{x}}, \end{array}$$

where *P*_*i*_ is the percentage composition, *W*_*i*_ is the weight of individual components, and *W*_*x*_ is the weight of total sampled waste.

### 2.8. Data Analysis

The study analyzed data from questionnaires and color-coded bins using the Special Package for Social Sciences (SPSS) Model 22, categorized and coded manually. A sample *t*-test was used to compare waste fractions in bins. Findings were illustrated using frequencies and percentages.

## 3. Results

### 3.1. Demographics

The demographics captured in the survey included the occupations of the participants and the current level of their relative occupations.

### 3.2. Occupations of the Participants

The occupations captured in the study included administrators, lecturers, other workers on the campus (bankers, stationery workers, architects, among others in the commercial area), students, and cleaners. Out of a sample size of 499 (100%), administrators consists 8.0%, lecturers 8.2%, workers 14.4%, students with the absolute majority 59.9%, and cleaners 9.4% in Table 1.

### 3.3. The Current Level of the Participants

Additionally, the current levels of each of the participants were categorized into year one, year two, year three, year four, university staff, or others. The first four (4) categories applied to the students, and others to the other occupations (workers, administrators, lecturers, and cleaners).

The students were the highest inhabitants on campus, representing 59.5%, with the other category being 40.5% in Table 2.

### 3.4. Information on Waste Segregation

Assessing the university community's awareness of waste segregation on campus, it was realized that 56.1% were privy to the waste segregation system on campus and 43.9% had no idea such a system was operating on campus in Table 3.

### 3.5. Channel of Information

**Figure 3:**
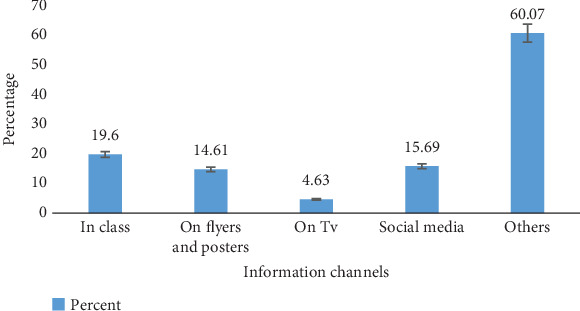
Channels for spreading information on waste segregation.

Of those participants who heard about the ongoing waste segregation on campus, 19.6% were privy to the information through class interactions, 14.61% of the participants knew about the system through floated flyers and posters, and a further 4.63% became aware of the segregation system through television advertisement, 15.69% became aware of segregation through social media platforms, and a majority of 60.07% came by this information from other sources not captured in the questionnaires such as friends, at the halls, campus church, and in the school shuttles dawn broadcasting in Figure 3.

### 3.6. Practice of Segregation

**Figure 4:**
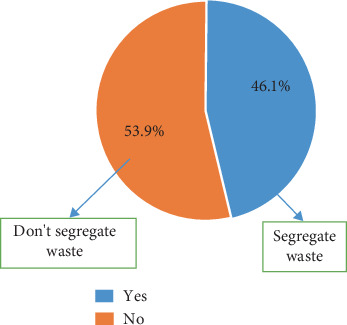
Practice of segregation on KNUST campus.

**Figure 5:**
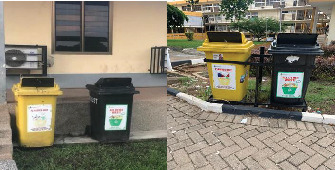
Picture of the bins used for segregation on campus.

On the KNUST campus, 46.1% practice waste segregation, and the remaining 53.9% do not practice waste segregation (Figures 4 and 5).

### 3.7. Types of Wastes Generated

**Figure 6:**
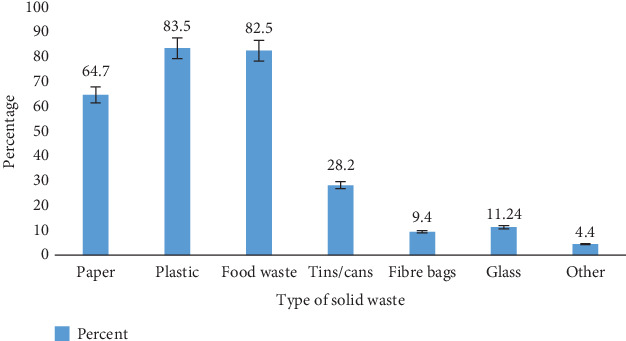
Types of waste generated on the KNUST campus.

Analysis of the waste components generated on campus revealed that 64.7% of generated paper was part of the total waste they produced, a substantial 83.5% generated plastics, 82.5% generated food waste, and 28.2% generated tins/cans (Figure 6). On campus, 9.4%, 11.24%, and 4.4% of generated fiber bags, glass, and other waste types were generated, respectively.

### 3.8. Notice of Segregation Bins

**Figure 7:**
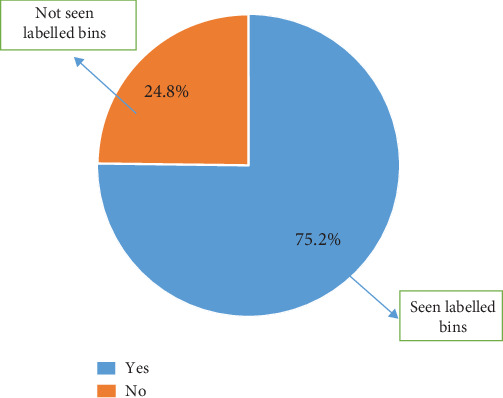
Notice of waste segregation bins on campus.

Of the participants, 75.2% on campus concurred that they had seen the bins on campus, while 24.8% concurred that they had not noticed the well-labeled coded bins placed around campus (Figure 7)

### 3.9. Reason for Introduction of Bins

**Figure 8:**
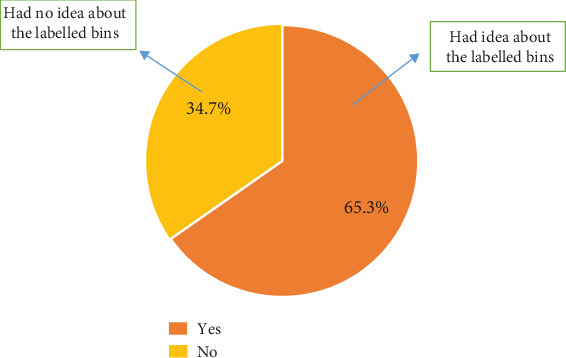
Reason for the introduction of segregation bins.

Further probing revealed that 65.3% of the participants who noticed the introduction of well-labeled and color-coded bins on campus knew why they had been placed around. Then, 34.7% had no idea why the well-labeled and color-coded bins were placed around campus (Figure 8).

### 3.10. Knowledge About Color Codes

**Figure 9:**
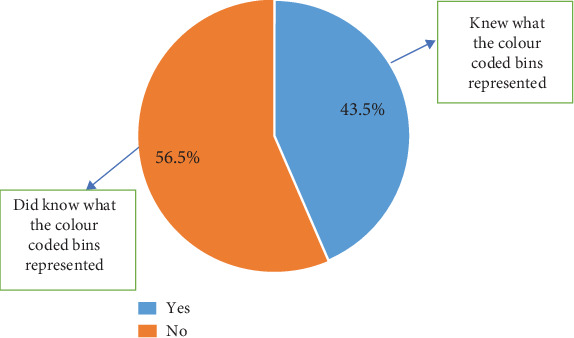
Knowledge about color codes on bins.

The study further revealed that 43.5% knew what the color codes of the bins represented. The majority, 56.5%, did not have any knowledge of what color codes the bins represented (Figure 9).

### 3.11. Level of Adherence to Waste Segregation

**Figure 10:**
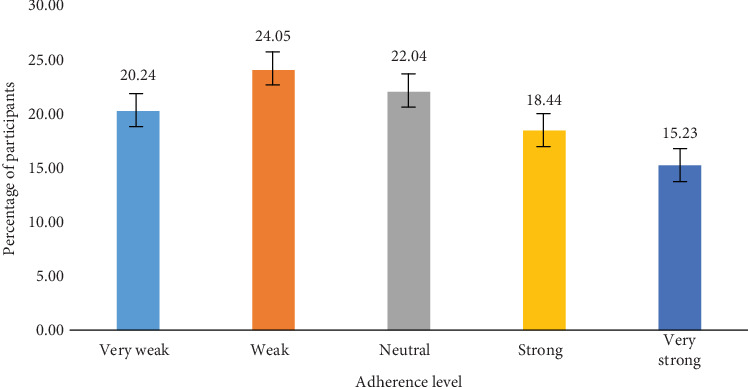
Level of adherence to segregation instructions on bins.

Of the participants, 20.24% adhered very weakly to the segregation instructions on the bins, 24.05% adhered weakly, 22.04% were neutral concerning their adherence, 18.44% concurred that they adhered strongly, and 15.23% also concurred that they adhered very strongly (Figure 10). This indicates that segregation on campus is not strictly adhered to, as indicated by the data.

### 3.12. Bin for Plastic Waste

**Figure 11:**
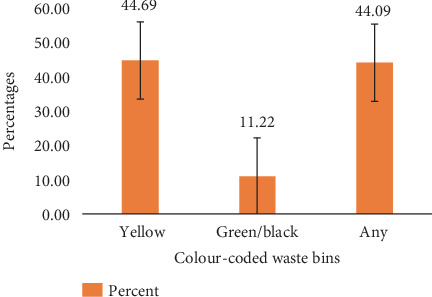
Bin for disposal of plastic waste.

The study also showed that 44.69% properly disposed of their plastic waste in the yellow-coded bins (bins designated for plastic waste). Then, 11.22% disposed of plastic wastes in green/black-coded bins (bins designated for all other wastes). Interestingly, 44.09% disposed of their plastic waste in any of the aforementioned bins, with their percentage almost at par with those who properly disposed of their waste (Figure 11).

### 3.13. Bin for All Other Waste

**Figure 12:**
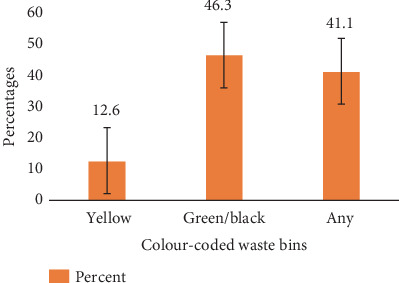
Bin for disposal of all other waste types.

Of the participants, 12.6% disposed of all other forms of waste in the yellow-coded bins and 46.3% disposed of all other forms of waste in the green/black-coded bins (bin designated for all other waste) (Figure 12). Interestingly, 41.1% disposed of all other forms of waste randomly in any of the segregation bins.

### 3.14. Agreement to Segregation

**Figure 13:**
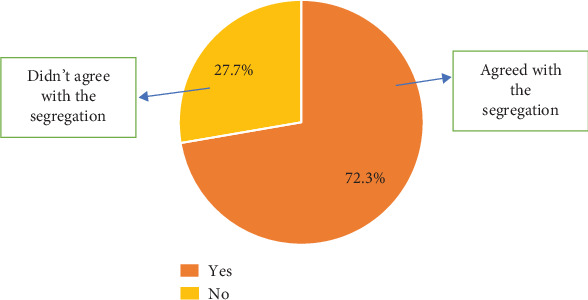
Agreement with the segregation system.

The findings of this study further revealed that 72.3% agreed with the solid waste segregation system as against 27.7% who expressed their disagreement with the segregation system (Figure 13).

### 3.15. Accuracy of Segregation

**Figure 14:**
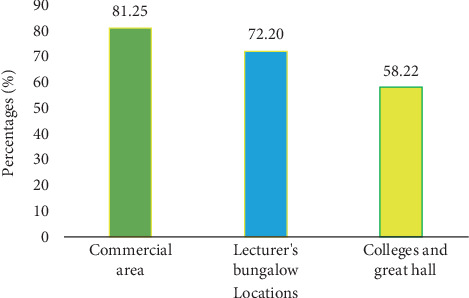
Mean segregation levels of plastics in different areas on campus.

The findings of the study revealed that the mean segregation level of plastic waste in the commercial area was the highest at 81.25%, followed by the lecturer's bungalow at 72.20%, with the lowest at 58.22% in the colleges and great hall areas (Figure 14).

**Figure 15:**
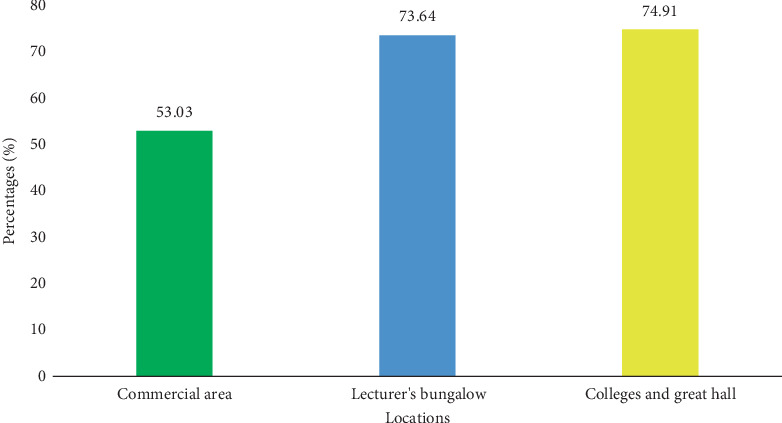
Mean segregation levels of all other waste types at different areas on campus.

Data collected in this study also revealed that the mean level of segregation of all other wastes was highest in the college and great hall regions at 74.91%, followed by the lecturer's bungalow with 73.64%, with the least being the commercial area at 53.03% (Figure 15).

### 3.16. One-Sample Test

One sample *t*-test was performed to compare the overall mean percentage of plastic waste and all other wastes in the appropriate color-coded bin after segregation against an ideal segregation percentage (100). The mean overall percentage of plastic waste in the yellow-coded bins (*M* = 69.3616, SD = 32.65373) was significantly less than the ideal segregation percentage; *t* (32) = −5.390, *p* < 0.005 (Table 4).

The overall mean percentage of all other waste in the green/black-coded bins (*M* = 72.6959, SD = 58.10382) was significantly less than the ideal segregation percentage; *t* (32) = −2.699, *p* = 0.011 (Table 5). The alpha value presents *p* < 0.05.

### 3.17. Plastic Waste at the Commercial Area

The plastic waste segregated at the commercial area has two of the seven types of plastics namely, PET and LDPE. The total weight of plastics collected was 6.5 kg. The PET was more than the LDPE by weight, that is, 5 and 1.5 kg, respectively (Table 6).

### 3.18. Plastic Waste at the Lecturer's Bungalows

A total weight of 32.5 kg of plastics was collected at the lecturer's bungalows. Three of the seven types of plastics were recorded, namely, PET, HDPE and LDPE with weights 17.0, 8.7, and 9.5 kg, respectively (Table 7).

### 3.19. Plastic Waste at the Colleges and Great Hall

At the colleges, a total weight of 18.5 kg of plastics was collected. Three of the seven types of plastics were recorded, namely, PET, LDPE, and PS weighing 11.5, 4.1, and 2.9 kg, respectively (Table 8).

**Figure 16:**
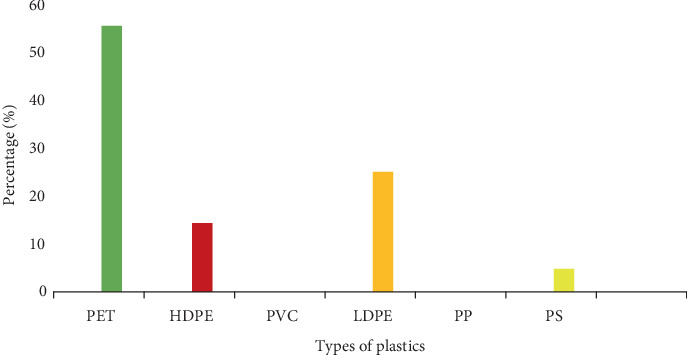
Summary of results of the composition of the different types of plastic in the KNUST waste stream.

There are different types of plastic composition in KNUST campus waste stream, and this indicates that PET is the highest plastic waste, while PVC and PP are not present in the waste stream (Figure 16).

## 4. Discussion

### 4.1. Demographic

As stated earlier, students make up the absolute majority of our study. This is justified by the fact that students comprise the absolute majority of all inhabitants of the KNUST campus, over 80,000, according to the acting university relations officer, Mr. Oberko, in 2021. Hence, the students were proportionally represented in our study. A critical look at the KNUST campus will reveal five principal categories of inhabitants based on their occupations: administrators, lecturers, cleaners, students, and other staff. This informed our choice of participants for the study.

### 4.2. Information on Waste Segregation

Information concerning solid waste segregation is not widespread on campus. This is evident in the 43.9% of our respondents who had no idea of the waste segregation system on campus. The segregation system was just set up on campus with the anticipation that inhabitants would understand it because of the labeling and separate solid waste appropriately. Also, new and old students in and out every academic year could affect the efficient segregation of waste on campus; however, the university could add it to the orientation for freshmen and women. Furthermore, there are no signs on campus indicating the practice of waste segregation on campus to inform any new member of the university to encourage them to also segregate their waste. This would have also kept new employees, visitors, and fresh students informed of the program [19]. Generally, publicity is low, especially among students who unfortunately make up the majority of the population. The reason could be that publicity channels are relatively inefficient in conveying the segregation message to the inhabitants. This is evident because 32.7% of the respondents, mostly lecturers, knew about the waste segregation system through other channels. These other channels included receiving messages from the waste department on campus.

Even though 75.2% concurred that they had seen the bins, this implies that the bin placement on campus is strategic; that is, the bins have been placed at locations that allow people to easily notice them. The majority of the participants, 56.5%, did not know what the color codes of the bins represented. This indicates that the level of segregation of waste in the bins is not principally based on knowledge but on sheer luck and randomness. This then indicates that the 65% of plastics in a yellow-coded bin recorded could be a result of luck rather than knowledge on the part of the one discarding the waste. The majority of the inhabitants do not (53.9%) practice waste segregation as expected from the university, as against 46.1% who segregate their waste [19, 21, 22]. This goes to validate further the claim that information concerning the ongoing waste segregation is not widespread. Even though the values obtained were not encouraging, this was a good system that, when encouraged, will help improve waste collection and value addition. The university needs to get more channels to inform the inhabitants of the program and its importance.

As analyzed earlier, 83.5% of the total participants generate plastic waste as a component of the total waste produced. Since a lot of plastic waste is generated on campus, if waste segregation is done properly, there will be a ready supply of plastic for the KNUST Recycling Plant. Also, 82.5% and 64.7% of food and paper waste, respectively, are disposed of on campus, indicating that composting can be produced by segregating organic waste from the other wastes [23]. This can be used as fertilizer for the greening of campus and packaged for sale to the public. This exposes a lapse in the current segregation system on the KNUST campus (i.e., plastic waste and all other waste). It is not a standard system and is not completely exploited since there are many other waste types on campus.

### 4.3. Level of Adherence to Waste Segregation

A Likert scale employed to show the level of adherence of participants to waste segregation on campus revealed that most inhabitants adhered weakly to the segregation instructions on the bins. It was determined that 44.69% disposed of their plastic waste in the designated waste bin and 56.31% disposed of their waste improperly; this indicates waste segregation on KNUST Campus is largely ineffective. Most people were aware of solid waste management strategies but did not put them into practice [24]. Then, 46.3% of our participants disposed of all other forms of waste correctly (i.e., in the green/black-coded bins). This goes to validate that some inhabitants have little or no idea that there was a waste segregation system in place. Most of the inhabitants do not know exactly why the bins were introduced and most of the inhabitants do not know what the color codes on the bins represent. Feng et al. [25] reported similarly that the distance between a residence to garbage collection facilities and village layout is negatively significant with rural residents' household solid waste disposal behavior, while local economic level and household educational level are positively significant with rural residents' solid waste disposal behaviors.

### 4.4. Plastic Waste Segregation

The small number of bins in the commercial area accounted for the high level of plastic waste (81.25%) in the yellow bins compared to the other locations. Ignorance on the part of students, who mostly dominate the college and great hall areas, accounted for the low level of plastic waste (58.22%) in the yellow bins compared to other locations. There has been a decrease in people's attitudes toward social commitment to participate in solid waste management, and this affects the accuracy of waste management [24]. Usually, students do not eat when they go to the faculty area, but for the commercial area, we have a mix of people with varying activities ongoing within the area. The high level of all other wastes (74.91%) in the green or black bin could be attributed to the high production of all other wastes aside from plastic on campus.

A low level of all other waste (53.03%) was obtained in the commercial area. However, the commercial area was expected to have high levels of other waste, like food waste and paper, in their green or black bins. This was because banks, stationery, restaurants, and other shops made the expectation of all other waste types to be high (i.e., paper). This indicates that the restaurant might have alternative waste disposal; the banks store the waste papers because they contain sensitive information.

The overall mean percentage of plastics in the yellow bin (bin designated for plastic waste) was 69.36% and that of all other waste in the green/black bin (bin designated for all other wastes) was 72.69%. Both were lower than the ideal assumed segregation level of 100% to help improve the value addition of solid waste. MSW collection and disposal is one of the major problems of the urban environment [23]. This implies that the segregation system on campus is largely ineffective. This could also be attributed to the fact that awareness of the segregation system, in general, is very low. In most communities around the world, people are much aware of the serious consequences of improper solid waste management practices but the negative attitude of implementation gives rise to chaotic situations [24].

Understanding the plastics' service life is crucial in the current climate. Disposable packaging has gained popularity in developing economies [26]. Due to insufficient waste management, this market uses substantial amounts of PET and polyolefin (LLDPE, PP, and HDPE), which end up in the environment. Although the material is the same, the service life of the packaging is reduced. This material might find a secondary market, and innovations in the field may open up new markets, employment prospects, and manufacturing opportunities, all of which add value [27]. These results demonstrate that as a community, KNUST generates the same type of plastic waste developing economies generate, as stated by Kosior and Crecenzi [28] and the spatial distribution of the types and weight of the plastic waste depends on the preferences of the population as well as the wealth of the population.

A total weight of 60.2 kg of plastic waste was recorded during the sampling period. It was discovered from the data recorded that KNUST's waste stream consists of four of the seven types of plastics: PET, HDPE, LDPE, and PS. PET weighed 33.5 kg, corresponding to 55.6% of the total plastic weight; HDPE weighed 8.7 kg, corresponding to 14.4%; LDPE weighed 15.1 kg, corresponding to 25.08%; and PS weighed 2.9 kg, corresponding to 4.8% (Figure 15). It was also discovered that the bungalows generated a greater portion of the weight of plastic waste (32.5 kg) which corresponds to 53.9% of the weight of plastic waste in the entire campus. This indicates that if the waste is properly segregated, the recycling plants can get a lot of plastic materials for recycling and, hence, add to internally generated funds of the university.

At the commercial area, PET and LDPE were the plastics waste sorted from the waste bins with weight percentages of 76% and 23%, respectively (Table 6). This was because the commercial area is made up of banks, restaurants, markets, shops, and all kinds of people patronize the place and which makes place very busy. Usually, the people that visit the place go there to transact business or buy. Because of the nature of the place, people stay there for a shorter period and the openness of the place causes an increase in temperature, which cause people to drink a lot of water. This could be one of the reasons for the types of plastics identified in the area.

The lecturer's bungalows produced the highest amount of plastic waste at 32.5 kg; this is because most of the bins used for the segregation are situated there and the bungalows have the highest number of permanent population as compared to the commercial areas and colleges where people come and go.

PET was found to weigh 48.3%, LDPE 26.99%, and HDPE 24.7% in the lecturer's bungalows (Table 7). The most widespread polymers in use are PET, HDPE, LDPE, PVC, PP, and PS [29]. In comparison to the commercial sector, the waste stream from bungalows contained HDPE, which is mostly utilized for things like detergent bottles, shampoo bottles, and other such items. This is because households frequently use goods like shampoos, detergents, and items made of HDPE. The lecturers produced a higher amount of PET plastics which are associated with high-end products such as plastic bottles for water, soft drinks, etc. which shows a sense of affluence as compared to relatively lesser-end products associated with LDPE plastics.

At the colleges, PET and LDPE were present weighing 62% and 22%, respectively, with the introduction of PS weighing 15% (Table 8). The smallest amount of plastics was recorded here weighing 18.5 kg. This is because the bins were sparsely distributed, and also, the area is associated with students moving to and fro from lectures, so there is little or no consumption of plastic packaged products unless around the few canteens dispersed in the area. The introduction of PS here as compared to the other data sites is due to the presence of canteens, where students visit to eat in between class hours and consume food in PS take-away containers.

## 5. Conclusion and Future Perspective

It can be concluded that waste segregation on KNUST Campus is largely ineffective, evident in the fact that average levels of particular waste types in appropriate waste bins are lower compared to the population. The inhabitants generally have a poor attitude towards the waste segregation system on campus because of ignorance of what the system entails. Also, ignorance and low level of monitoring are the principal driving forces behind the system's failure to achieve maximum success in segregation. Also, significant percentages of inhabitants produce food waste (82.5%) and paper waste (64.7%), making it necessary to expand the segregation system to capture organic waste and other waste types. The plastic waste obtained from the solid waste stream was also categorized into PET, HDPE, LDPE, PVC, PP, PS, and other plastics. Across the campus, it was revealed that four (PET, HDPE, LDPE, and PS) of the seven types of plastics were discovered to be dominant in the waste stream. PET and LDPE representing plastic bottles mostly and sachet rubbers were observed to be relatively higher than the others. Composting becomes a good option to adopt. Other waste types, such as tins/cans and glass, can also be recycled or sold to recycling firms for extra income. Moreover, KNUST's plastic waste stream represents a resource reserve that can be used to boost revenue to augment internally generated fund.

If not for lack of funds, the research would have been carried out using the entire bins in the university.

To enhance the effectiveness of waste segregation on KNUST Campus, publicity needs to be intensified especially within the student body. Appropriate channels should also be adopted to convey the message of waste segregation. A composting facility should be introduced on campus for the recycling of organic waste. Segregation should be more diversified. The segregation system should be expanded to cater for other waste types, prominently organic waste because food waste can be used to generate compost. The number of segregation bins should be increased especially around the halls to generate more plastic raw materials for the recycling facility. Futher study can be carried out on a gated community segregation system to ascertain the level of efficiency to inform strategic to put in place should there be possible role at in a whole community.

## Figures and Tables

**Table 1:** Occupations of participants on the KNUST campus.

**Category**	**Frequency**	**Percentage (%)**
Administrators	40	8.0
Lecturers	41	8.2
Worker	72	14.4
Students	299	59.9
Cleaners	47	9.4
Total	499	100.0

**Table 2:** Participants on the KNUST campus.

**Category**	**Frequency**	**Percentage (%)**
Year one	50	10.0
Year two	65	13.0
Year three	46	9.2
Year four	136	27.3
University staff/others	202	40.5
Total	499	100.0

**Table 3:** Information on waste segregation.

**Response**	**Frequency**	**Percent (%)**
Yes	280	56.1
No	219	43.9
Total	499	100.0

**Table 4:** One-sample test to compare mean levels of segregation to an ideal segregation level.

	**Test ** **v** **a** **l** **u** **e** = 100					
	**T**	**d** **f**	**Sig. (2-tailed)**	**Mean difference**	**95% confidence interval of the difference**	
					**Lower**	**Upper**
Percentage of plastic waste in the yellow bin	−5.390	32	0.000	−30.63840	−42.2169	−19.0599
Percentage of all other wastes in all other waste bins	−2.699	32	0.011	−27.30413	−47.9068	−6.7014

**Table 5:** One-sample statistics concerning segregation levels.

	**N**	**Mean**	**Std. deviation**	**Std. error mean**
Percentage of plastic waste in the yellow bin	33	69.3616	32.65373	5.68428
Percentage of all other waste in green/black bin	33	72.6959	58.10382	10.11458

*Note*: The alpha value, *p* < 0.05.

**Table 6:** Plastic waste composition at the commercial area.

**Type of plastic**	**Total weight/day (kg)**	**Percentage (%)**
Polyethylene terepthalate	5.0	76.9
High-density polyethylene	0.0	0.0
Polyvinyl chloride	0.0	0.0
Low-density polyethylene	1.5	23.07
Polypropylene	0.0	0.0
Polystyrene	0.0	0.0

**Table 7:** Plastic waste composition at the bungalows.

**Type of plastic**	**Total weight/day (kg)**	**Percentage (%)**
Polyethylene terepthalate	17.0	48.3
High-density polyethylene	8.7	24.7
Polyvinyl chloride	0.0	0.0
Low-density polyethylene	9.5	26.99
Polypropylene	0.0	0.0
Polystyrene	0.0	0.0

**Table 8:** Plastic waste composition at the colleges and great hall.

**Type of plastic**	**Total weight/day (kg)**	**Percentage (%)**
Polyethylene terepthalate	11.5	62.162
High-density polyethylene	0.0	0.0
Polyvinyl chloride	0.0	0.0
Low-density polyethylene	4.1	22.162
Polypropylene	0.0	0.0
Polystyrene	2.9	15.675

## Data Availability

Data are available upon request from authors.

## Questionnaire for Participants

This is a study being conducted by final year Environmental Science Students of KNUST and it is aimed at assessing the effectiveness of waste segregation on the KNUST campus. We would like to assure you that your identity will remain anonymous and your responses will also be treated with confidentiality. To assist us with this study, we humbly request that you take time out of your busy schedule to answer the following questions.

Please tick the option that applies to you:

1. Student ()
2. Lecturer ()
3. Administrator ()
4. Others (Please Specify) _________________
5. If Student, What Level?

100 ().

200 ().

300 ().

400 ().

Others (Please specify ____________________).

6. Have you heard about waste segregation ongoing on campus?

Yes ().

No ().

7. If yes, how did you hear about it?

In Class ().

On TV ().

Social media ().

On flyers and posters ().

Others ().

8. Do you practice waste segregation in your hall/hostel/home/office?

Yes ().

No ().

9. What type of solid waste do you mostly generate in your hall/hostel/home/office?

Paper ().

Plastic ().

Food Waste ().

Tins or Cans ().

Fiber Bags ().

Glass ().

Other ().

10. Have you noticed that waste bins have been placed around campus?

Yes ().

No ().

11. Do you know why they have been placed all around campus?

Yes ().

No ().

12. Do you know what the colors of the bins represent?

Yes ().

No ().

13. How well do you adhere to the waste segregation instructions on the bins on campus on a scale of 1–5? (with 1 being very weak and 5 being very strong)

1 ().

2 ().

3 ().

4 ().

5 ().

14. Which bin do you put your plastic waste in?

Yellow ().

Green/black ().

Any ().

15. Which bin do you put all other types of waste that aren't plastic in?

Yellow ().

Green/black ().

Any ().

16. Do you agree with the waste segregation being practiced on campus?

Yes ().

No ().

If Yes/No why?.................................................................................................................

17. Do you have any other comments concerning the waste segregation ongoing on campus?
